# Supplementary material for: Scalable nano-architecture for stable near-blackbody solar absorption at high temperatures
Source: Nat Commun. 2024 Jan 9;15:384. doi: 10.1038/s41467-023-44672-3 (PMC10776863; doi:10.1038/s41467-023-44672-3)
Supplement: Supplementary file 1 — Supplementary Information [file 41467_2023_44672_MOESM1_ESM.pdf]

Supplementary Information

Scalable nano-architecture for stable near-blackbody solar absorption at high temperatures

Yifan Guo,[1,2] *Kaoru Tsuda,*[3] *Sahar Hosseini,*[1,2] *Yasushi Murakami,*[4] *Antonio Tricoli,*[5,6] *Joe Coventry,*[2] *Wojciech Lipiński*[7] *and Juan F. Torres*[*1]

[1]ANU HEAT Lab, School of Engineering, Australian National University, Canberra, Australia. [2]Thermal Energy Group, School of Engineering, Australian National University, Canberra, Australia. [3]Nano Frontier Technology, Tokyo, Japan. [4]Faculty of Textile Science and Technology, Shinshu University, Ueda, Japan. [5]Nanotechnology Research Laboratory, Faculty of Engineering, University of Sydney, Sydney, Australia. [6]Nanotechnology Research Laboratory, Research School of Chemistry, Australian National University, Canberra, Australia. [7]The Cyprus Institute, Nicosia, Cyprus. [*]e-mail: felipe.torres@anu.edu.au

Supplementary Figures

Fig. 1 Optical properties of materials in computational electromagnetics (CEM) modelling . 2
Fig. 2 CEM modelling: optical effect of nanosphere polydispersity 2
Fig. 3 CEM modelling: optical effect of nanosphere size 3
Fig. 4 CEM modelling: optical effect of nanosphere coverage 4
Fig. 5 CEM modelling: optical effect of ellipsoidal nanoparticles 5
Fig. 6 Independence study of calculation domain and polarisation for random configuration . 5
Fig. 7 Nanolayer effectiveness as a function of nanosphere diameter and matrix thickness . . 6
Fig. 8 Nanolayer effectiveness for other materials 6
Fig. 9 SEM analysis of effective nanosphere size and polydispersity 7
Fig. 10 SEM analysis of nanolayer coverage on coral-structured coating 8
Fig. 11 CEM model of monodisperse nanospheres with random configuration 8
Fig. 12 Vertical projection of nanospheres with a diameter of 100 nm 9
Fig. 13 Measured spectral optical effect of the nanolayer on a coral-structured coating 9
Fig. 14 Measured spectral absorptance with and without nanolayer 10
Fig. 15 Verification and validation of CEM modelling 13
Fig. 16 Photos of drone-assisted deposition experiment: procedure and analysis 15
Fig. 17 Nanolayer on absorber and spectral emissive power with and without nanolayer . . . 18

Supplementary Tables

Table 1 Effect of nanolayer on photo-thermal energy conversion efficiency 20

Supplementary Notes

Note 1 Verification and validation of the nanolayer CEM modelling 11
Note 2 Scalability experiment for drone-assisted nanolayer deposition 14
Note 3 High-temperature thermal emittance measurements 17

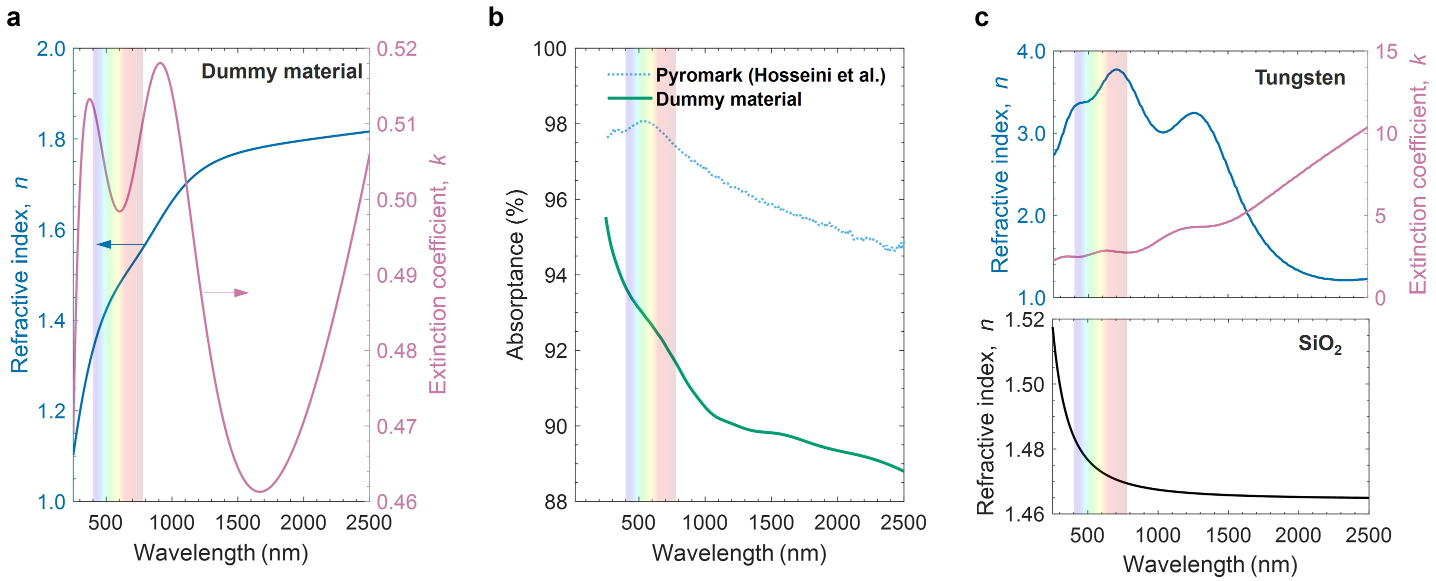

Supplementary Figure 1 | Optical properties of materials in computational electromagnetics (CEM) modelling. We employ a finite-difference time-domain (FDTD) method to model light–matter interaction. **a,** Complex refractive index of a modelled cutting-edge absorber or 'dummy' material. **b,** Spectral absorptance of the dummy material. The spectral absorptance of recently optimised coating of Pyromark 2500 [1] is included here as the reference. Note that the reflectance of the dummy material is still relatively high because it is a theoretical result based on the Fresnel equations for a perfectly flat surface (Supplementary Note 1), whereas the results for Pyromark are experimental and for a highly porous composite. **c,** Complex refractive index of tungsten [2] (top) and refractive index of silica [3] (bottom) whose extinction coefficient is so small that it can be neglected.

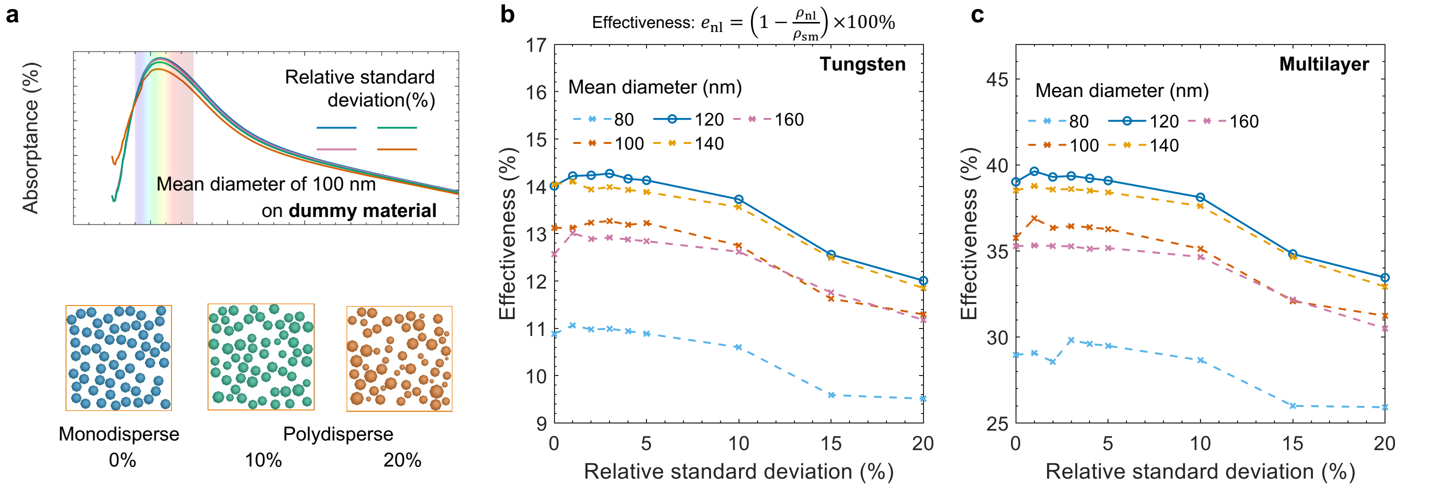

Supplementary Figure 2 | CEM modelling: optical effect of nanosphere polydispersity. a, Spectral absorptance of polydisperse nanospheres with a mean diameter of 100 nm and different relative standard deviations. The relative standard deviation is the standard deviation normalised against its mean value and expressed in percentage. Representative distributions for monodisperse and polydisperse nanospheres are shown. **b,** Effectiveness as a function of relative standard deviation for the nanospheres with polydisperse distribution on tungsten. **c,** Effectiveness of polydisperse nanospheres on a multilayer absorber.

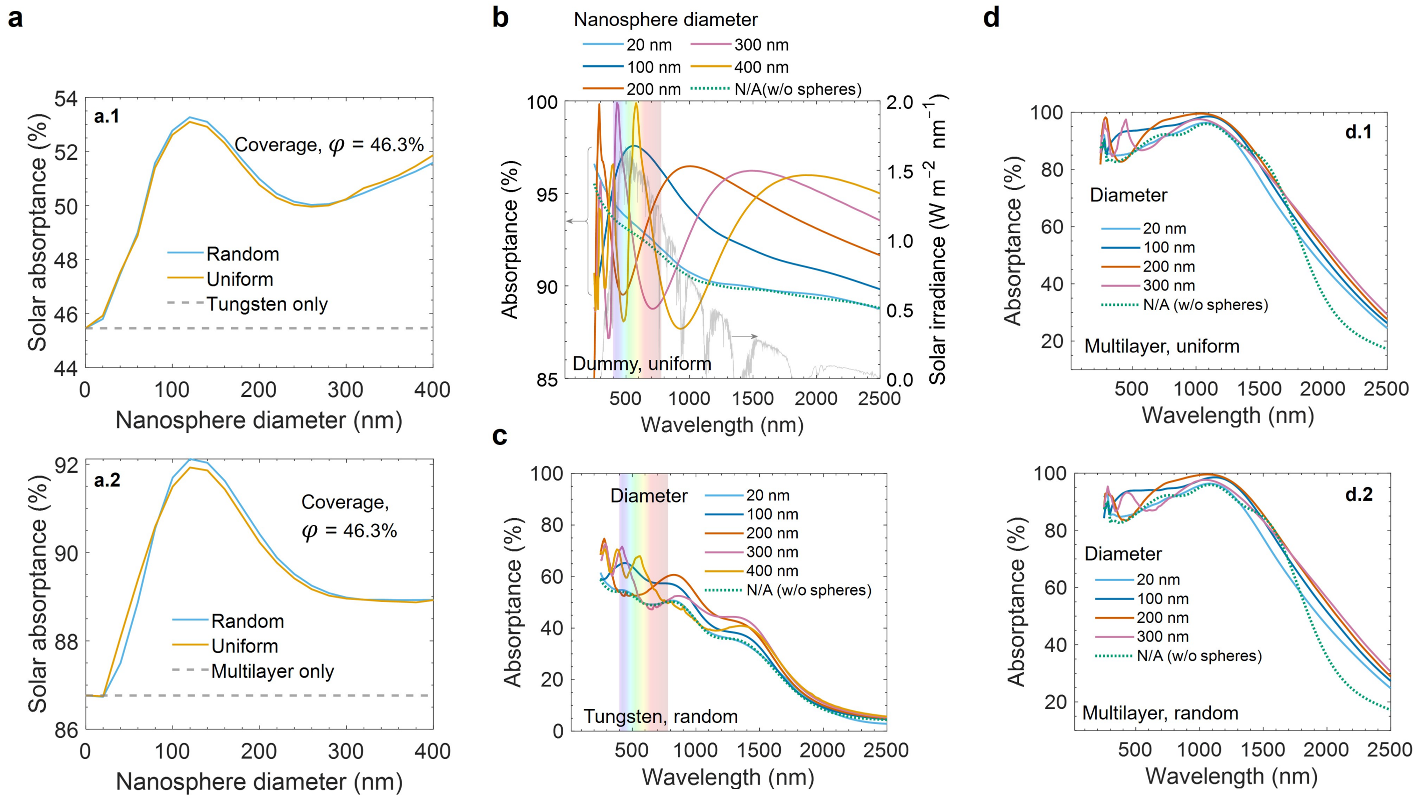

Supplementary Figure 3 | CEM modelling: optical effect of nanosphere diameter size. Nanolayers with a monodisperse nanosphere distribution and without matrix are considered together with two nanosphere distribution configurations: random as per inset of upper panel in Fig. 2a and uniform as per inset of lower panel in Fig. 2a. **a,** Solar absorptance as a function of nanosphere diameter with the configurations indicated in the legend for 46% coverage on two underlying absorbers: tungsten (a.1) and a multilayer composite (a.2). **b,** Spectral absorptance for different nanosphere diameters indicated in the legend on the dummy material with uniform configuration. **c,** Spectral absorptance of nanospheres on tungsten with a random configuration. **d,** Spectral absorptance of nanospheres on the multilayer composite with uniform configuration (d.1) and random configuration (d.2).

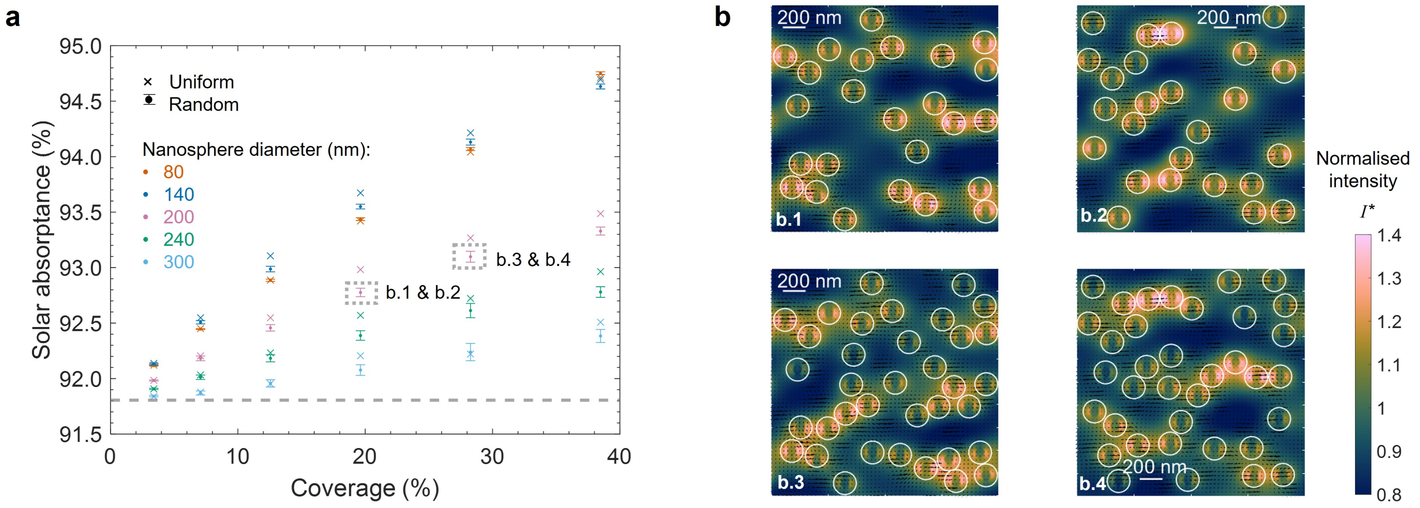

c

Diameter (nm) \ Coverage (%)	38.48		28.27		19.63		12.56		7.07		3.14	
80	94.752±0.013	94.713	94.069±0.014	94.041	93.437±0.013	93.420	92.886±0.010	92.887	92.446±0.004	92.451	92.117±0.002	92.121
100	94.993±0.013	94.987	94.306±0.017	94.294	93.624±0.016	93.628	93.005±0.017	93.032	92.499±0.007	92.521	92.110±0.004	92.115
120	94.870±0.017	94.901	94.257±0.022	94.288	93.598±0.018	93.657	92.977±0.023	93.062	92.458±0.011	92.499	92.054±0.005	92.058
140	94.632±0.022	94.695	94.131±0.027	94.214	93.550±0.021	93.673	92.987±0.025	93.104	92.508±0.015	92.548	92.133±0.006	92.137
160	94.183±0.027	94.289	93.796±0.034	93.939	93.310±0.027	93.487	92.826±0.027	92.978	92.411±0.019	92.445	92.087±0.006	92.092
180	93.725±0.032	93.854	93.431±0.041	93.605	93.035±0.033	93.234	92.638±0.029	92.773	92.295±0.021	92.323	92.035±0.006	92.038
200	93.329±0.036	93.487	93.098±0.049	93.268	92.776±0.038	92.982	92.457±0.029	92.548	92.182±0.021	92.205	91.983±0.005	91.984
220	93.014±0.042	93.211	92.827±0.057	92.981	92.561±0.042	92.757	92.305±0.030	92.379	92.089±0.020	92.112	91.942±0.006	91.941
240	92.780±0.048	92.964	92.613±0.064	92.722	92.388±0.043	92.570	92.183±0.032	92.232	92.012±0.020	92.033	91.907±0.006	91.907
260	92.606±0.052	92.776	92.451±0.070	92.487	92.256±0.045	92.421	92.088±0.033	92.122	91.954±0.020	91.966	91.881±0.007	91.880
280	92.480±0.056	92.634	92.334±0.075	92.361	92.159±0.047	92.317	92.018±0.033	92.021	91.909±0.019	91.934	91.860±0.007	91.857
300	92.384±0.059	92.508	92.239±0.078	92.221	92.077±0.048	92.206	91.957±0.033	91.953	91.870±0.018	91.878	91.842±0.007	91.839

Solar absorptance (%), random (yellow)
Solar absorptance (%), uniform (green)

Supplementary Figure 4 | CEM modelling: optical effect of nanosphere coverage. The dummy material is considered as the underlying solar absorber. **a,** The difference in terms of solar absorptance between uniform and random configuration varied with nanosphere diameter and coverage. **b,** The magnitude of the normalised light intensity (normalised against the incident power) from a top view at a plane just under the nanospheres and the wavelength of 860 nm for the chosen case (highlighted square in a). **c,** The comparison of solar absorptance for different coverage and nanosphere diameter with random and uniform configurations, and the error of random configuration introduced by the 20 different arrangements.

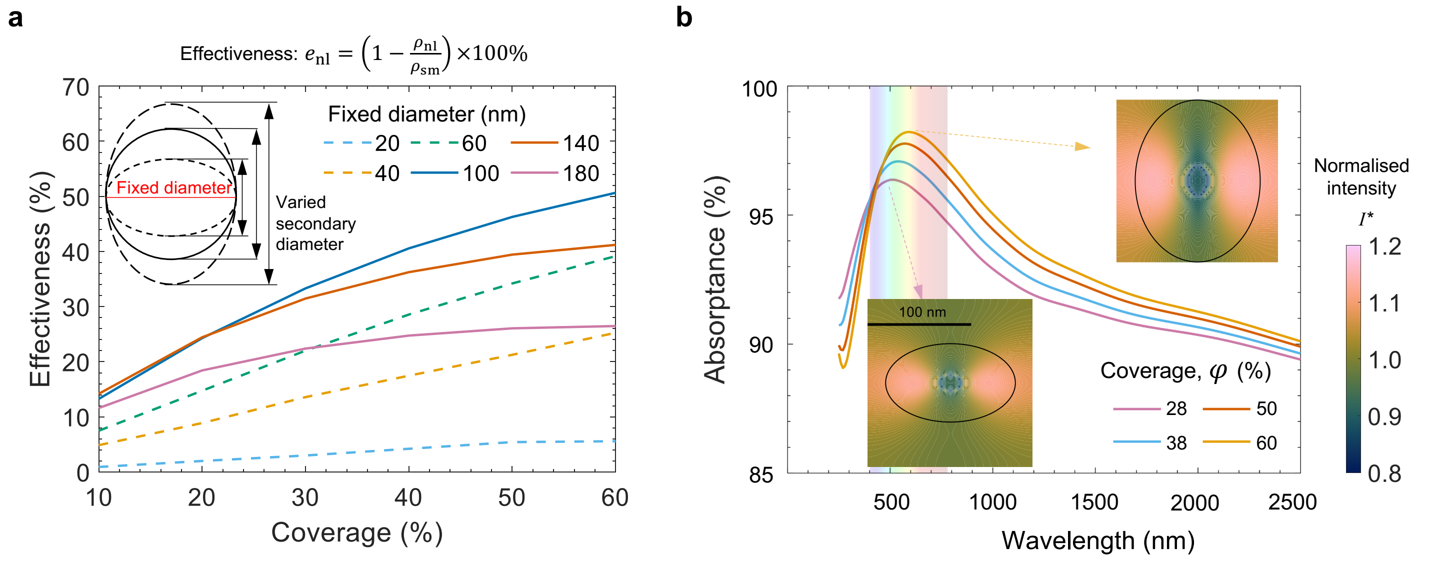

Supplementary Figure 5 | CEM modelling: optical effect of ellipsoidal nanoparticles. a, The effectiveness for uniform configuration as a function of the coverage for different secondary diameters on dummy material. The inset shows the schematic diagrams of nanoparticles with ellipsoidal shape, with the fixed diameter D_1 and varied secondary diameter D_2. The simulation setup is the same as in Supplementary Figure 12b. The coverage is calculated by $\frac{\pi D_1 D_2}{4A}$, where A is the total solar absorber area within one periodic calculation domain. **b,** Spectral absorptance of the absorbing material covered nanoparticles with fixed diameters of 100 nm with uniform configuration for coverage (by varying secondary diameters). The insets show the magnitude of the normalised light intensity just underneath the nanoparticles with the wavelength of 520 nm for a coverage of 28% and 600 nm for a coverage of 60%.

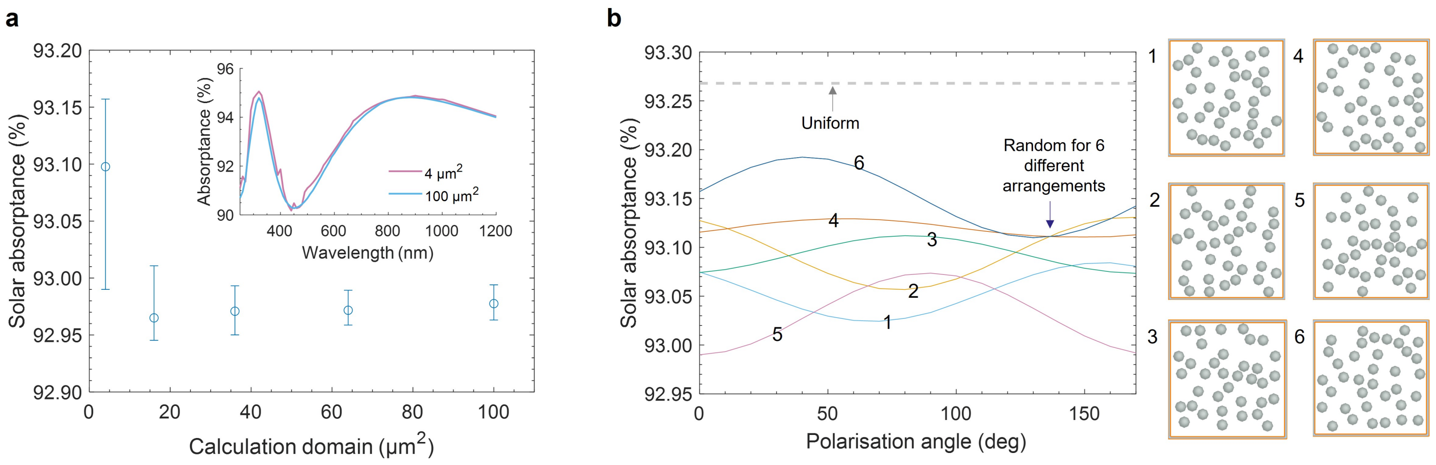

Supplementary Figure 6 | Independence study of calculation domain and polarisation for nanospheres with random configuration. The 200 nm diameter and 28.6% coverage of nanospheres on dummy absorber. **a,** The solar absorptance as a function of calculation domain of 20 arrangements in random configurations. The inset shows the spectral absorptance of different calculation domains. **b,** The solar absorptance as a function of polarisation angle. Zero polarisation angle is defined as the direction of the electric field parallel to the x axis in Supplementary Figure 12.

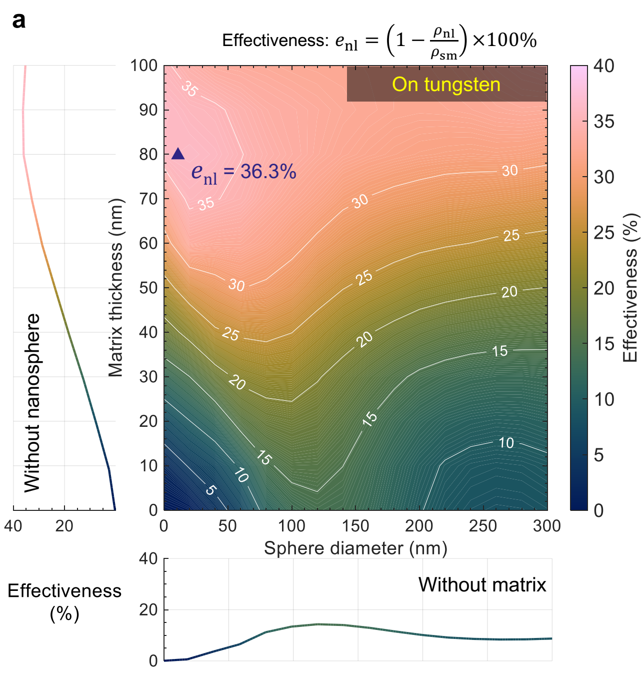

Supplementary Figure 7 | Nanolayer effectiveness as a function of nanosphere diameter and matrix thickness. Effectiveness when the silica nanolayer is placed on **a,** tungsten and **b,** multilayer composite. The nanospheres are located on the top of the matrix without immersion. Here, the nanospheres have a random configuration.

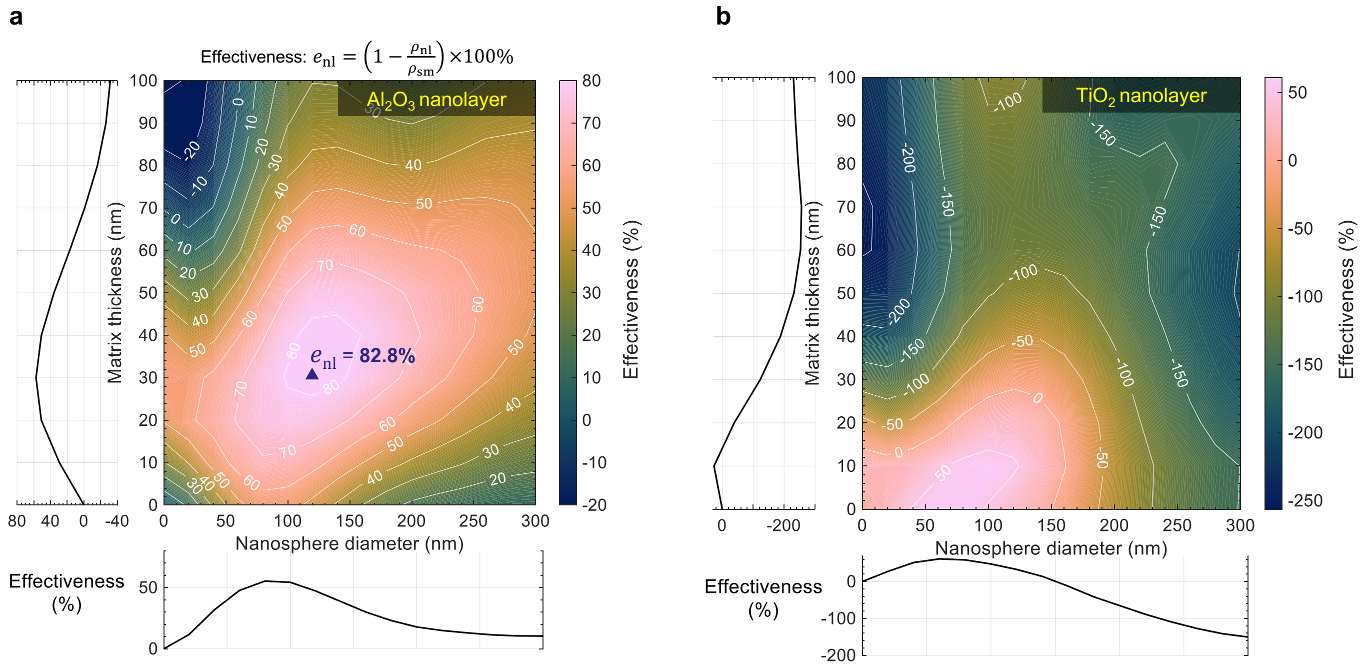

Supplementary Figure 8 | Nanolayer effectiveness as a function of nanosphere diameter and matrix thickness for other materials. Effectiveness when the nanolayer is composed of **a,** Alumina Al_2O_3 and **b,** Titania TiO_2. The randomly placed nanospheres are on the matrix without immersion, and the underlying material is a dummy absorber.

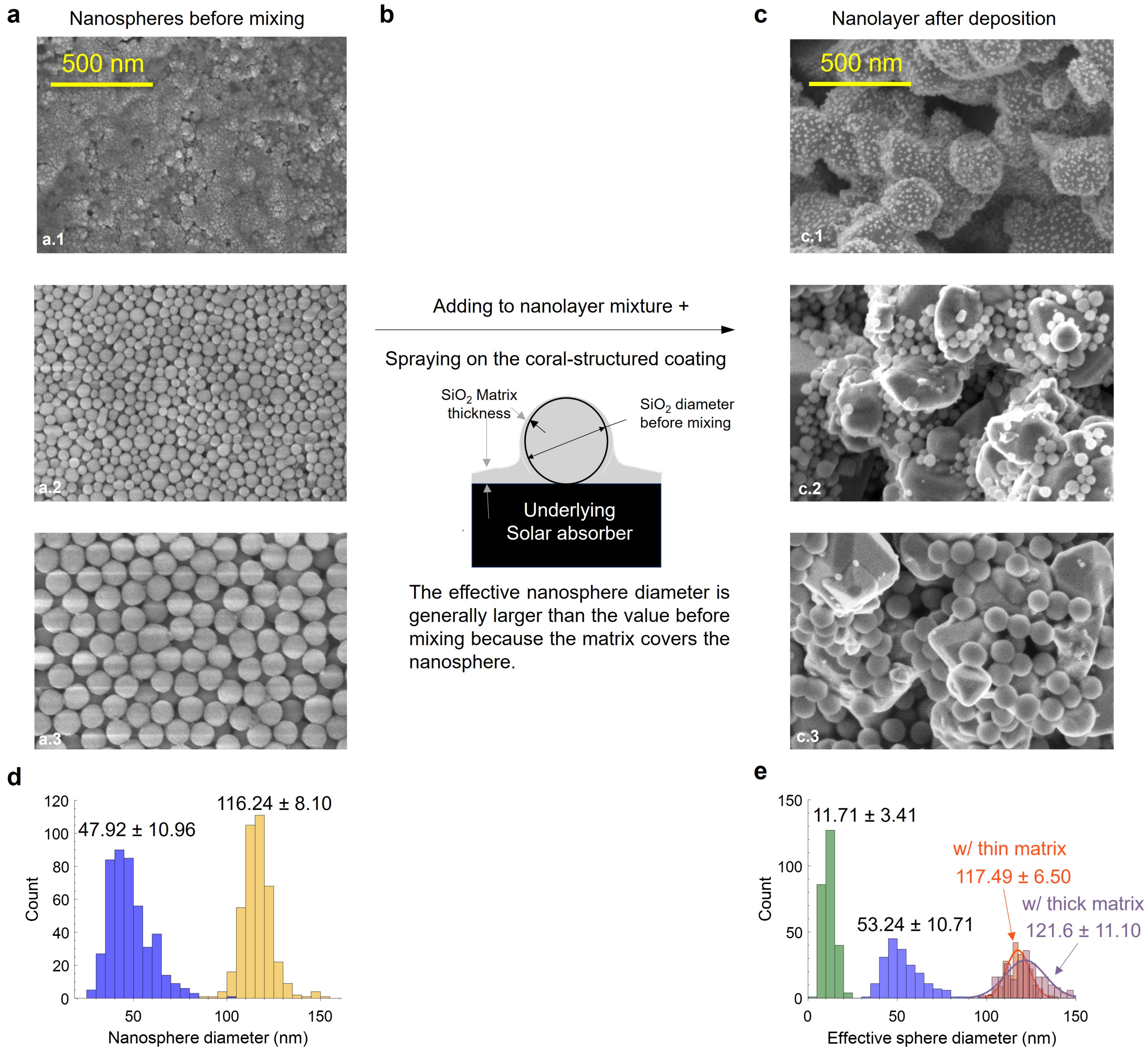

Supplementary Figure 9 | SEM analysis of effective nanosphere size and polydispersity. Nanospheres are sourced from different colloidal mixtures: IPA-ST (top SEM), IPA-ST-L (middle SEM), IPA-ST-ZL (bottom SEM). **a,** SEM images showing nanospheres before mixing in nanolayer mixture. **b,** Schematic diagram showing the side view of our layer nano-architecture focusing on a nanosphere. The matrix contributes to the effective nanosphere diameter after deposition on an underlying solar absorber. **c,** SEM images showing the nanolayer with thin matrix obtained from monomer TEOS deposited on a coral-structured coating. **d,** Nanosphere size distribution before mixing in the nanolayer mixture obtained from the SEM analysis in (a). A quantitative measurement for the nanospheres of ca. 11 nm in diameter is excluded due to large uncertainty. **e,** The count of effective nanosphere diameter (with arithmetic mean ± standard deviation) after deposition on a coral-structured coating obtained from the SEM analysis in Fig. 4e (thin matrix) and Fig. 5b (thick matrix).

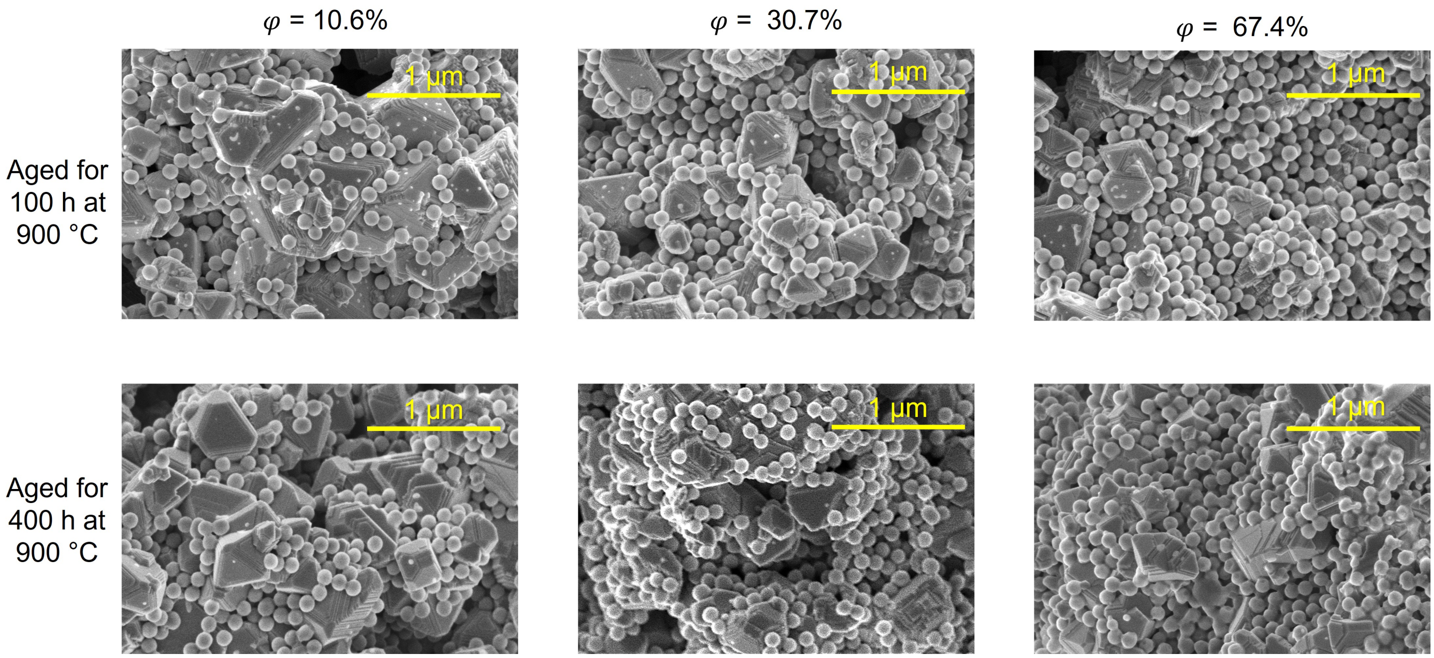

Supplementary Figure 10 | SEM analysis of nanolayer coverage on coral-structured coating. The SEM images are taken after ageing at 900 °C for 100 h (top) and 400 h (bottom). Note that the morphology of the underlying absorber consists of pigments of about 1 μm in sizes.

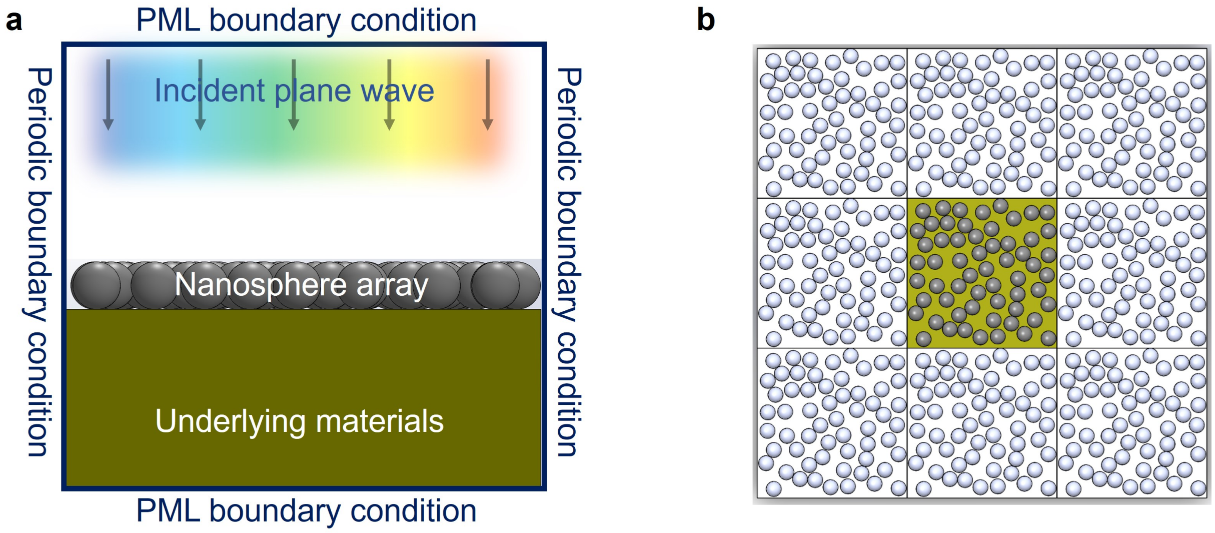

Supplementary Figure 11 | CEM model of monodisperse nanospheres with random configuration. a, Side view of simulation setup: the periodic boundary conditions are set along z on the lateral planes xz and yz; the perfectly matched layer (PML) boundary condition is set at the bottom and top xy plane boundaries. **b,** An example top view of a simulation setup: the brown area showing one periodic boundary (calculation domain) of nanospheres with random configuration.

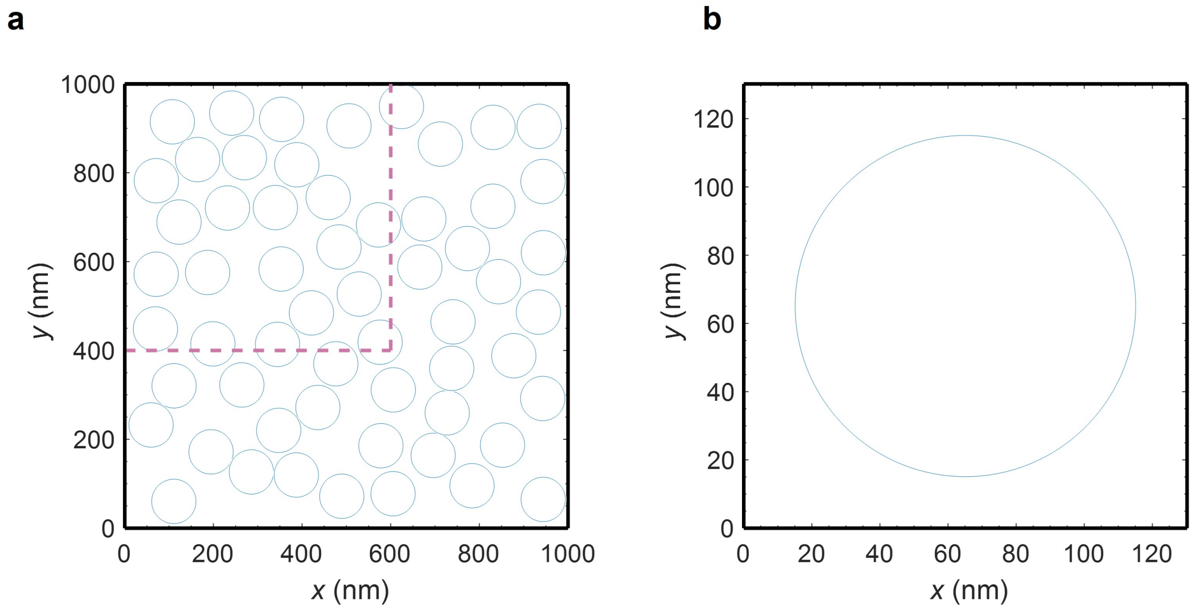

Supplementary Figure 12 | Vertical projection of nanospheres with a diameter of 100 nm. The boundaries of the calculation domain are periodic. Two configurations are shown. **a,** Random configuration within the area of 1 µm^2, which equals to 46% coverage. The square region delimited by dash lines indicates the area shown in the inset of Fig. 2e. **b,** Uniform configuration for the simulation region shown in the inset of Fig. 2d.

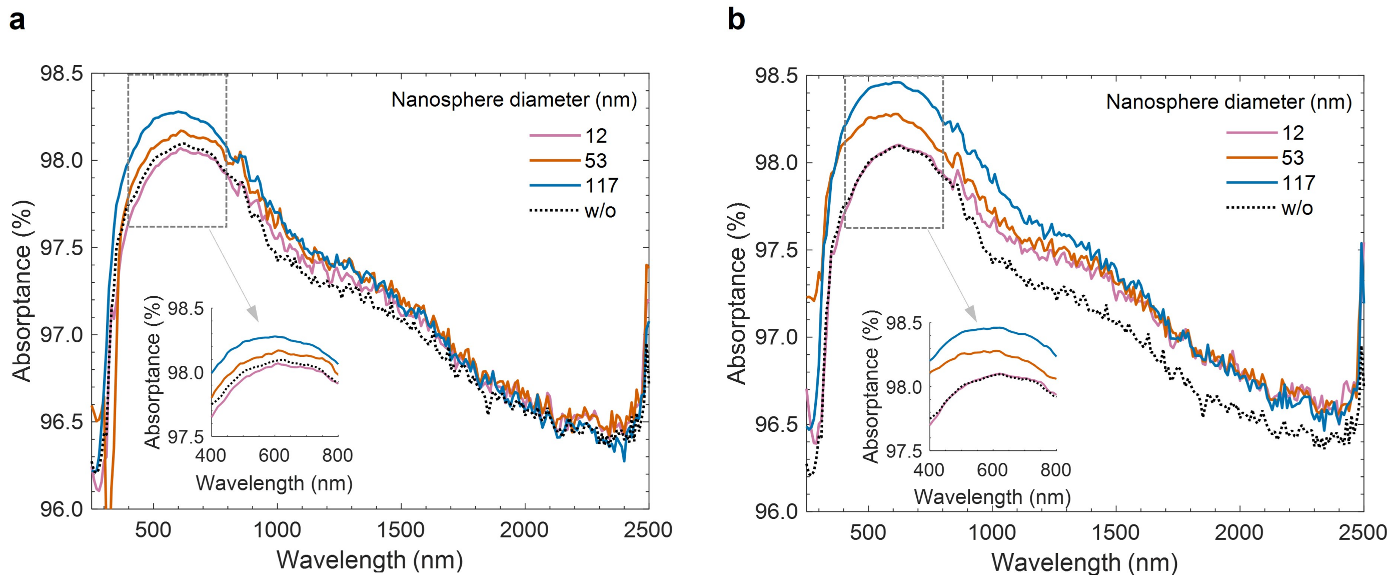

Supplementary Figure 13 | Measured spectral optical effect of the nanolayer on a coral-structured coating. The experimentally measured spectral absorptance shows the influence of nanospheres with different diameters for two nanosphere coverages in pristine condition. **a,** Coverage of 10.6%. **b,** Coverage of 67.4%.

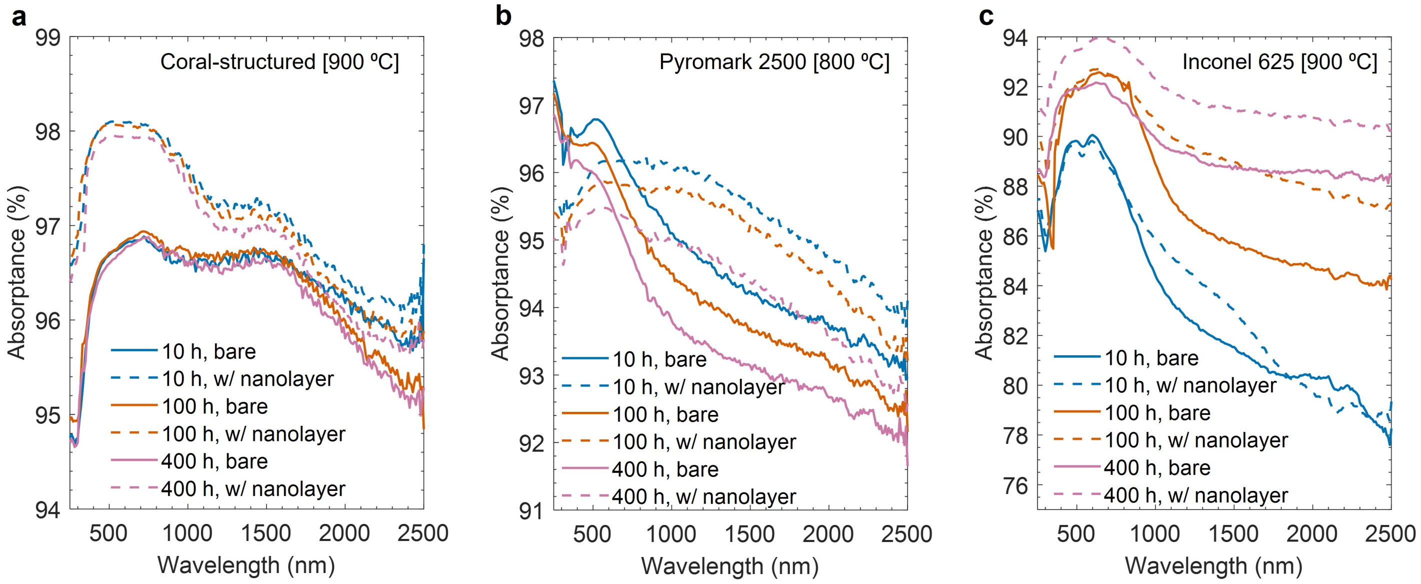

Supplementary Figure 14 | Measured spectral absorptance with and without nanolayer. The spectral absorptance without nanolayer is plotted with solid lines, whereas the absorptance with nanolayer is plotted with dashed lines. After ageing for 10 h (blue), 100 h (red) and 400 h (green) on different underlying absorbers. **a,** the coral-structured coating at 900 °C. **b,** Inconel 625 at 900 °C. **c,** Pyromark at 800 °C. These plots are reproduced from [4]. The calculated effectiveness is shown in Fig. 1e.

Supplementary Note 1:
Verification and validation of the nanolayer CEM modelling

We present an overview of the process of verification and validation for our computational electromagnetics (CEM) modelling *via* finite-difference time-domain (FDTD) method. The overall objective is to demonstrate the accuracy of FDTD models so that they may be used with confidence for nanolayer simulations and that the results be considered credible for decision-making in design. CEM modelling is conducted in Ansys Lumerical, a commercial photonics simulation package. First, the verification procedure compares CEM results with analytical solutions. Then, the validation procedure compares our numerical results with experimental results reported in the literature. Since this work is the first to report experimental results for nanolayers on materials of unknown optical properties (black spinels), here we use experimental results on multi-layer coatings reported in [5].

When a plane, time-harmonic electromagnetic wave travelling through a homogeneous film along the stratification direction of z, the characteristic matrix for solving the differential equations (derived from Maxwell's equations) [6] is

$$\boldsymbol{M}(z) = \begin{bmatrix} \cos(k_0 nz\cos\theta) & -\frac{i}{\sqrt{\frac{\epsilon}{\mu}}\cos\theta}\sin(k_0 nz\cos\theta) \\ -i\sqrt{\frac{\epsilon}{\mu}}\cos\theta\sin(k_0 nz\cos\theta) & \cos(k_0 nz\cos\theta) \end{bmatrix}, \tag{1}$$

where

$$\begin{aligned} n^2 &= \epsilon\mu, \\ k_0 &= \frac{2\pi}{\lambda}. \end{aligned} \tag{2}$$

Here, n is the refractive index, ϵ is the dielectric constant of the material, μ is the magnetic permeability of the material, k_0 is the wave number in free space, λ is wavelength and θ is the angle of incidence. For three non-magnetic media ($\mu = 1$) with a homogeneous dielectric film situated between two homogeneous media as shown in Supplementary Fig. 15a. In the characteristic matrix,

$$m'_{11} = m'_{22} = \cos\beta, \tag{3}$$

where

$$\beta = \frac{2\pi}{\lambda} n_2 h \cos\theta_2. \tag{4}$$

Here, h is the thickness of the film, subscript 2 is for the dielectric film such as silica in our simulation. In the following equations, we use subscript 1 for air and subscript 3 for the absorber material with a large extinction coefficient ($k >> 1$) such as tungsten in our simulation.

The extinction coefficient in the simulated wavelength range (250 nm to 2500 nm) of silica is very close

to zero so it is set as zero in the simulation (Supplementary Fig. 1c). According to Fresnel equations, the reflection coefficient between a silica film and air r_{12} can be expressed as

$$r_{12} = \frac{n_1 \cos\theta_1 - n_2 \cos\theta_2}{n_1 \cos\theta_1 + n_2 \cos\theta_2}. \tag{5}$$

When calculating the reflection coefficient between the silica film and absorber material, the amplitude ratio ρ_{23} and phase change ϕ_{23} need to be considered

$$\rho_{23}^2 = \frac{(n_2 \cos\theta_2 - n_3)^2 + k_3^2}{(n_2 \cos\theta_2 + n_3)^2 + k_3^2}, \tag{6}$$

$$\phi_{23} = \arctan \frac{2k_3 n_2 \cos\theta_2}{n_3^2 + k_3^2 - n_2^2 \cos^2\theta_2}. \tag{7}$$

In terms of these expressions, the total reflection coefficient r of these three layers becomes

$$r = \frac{r_{12} + \rho_{23} e^{i(\phi_{23}+2\beta)}}{1 + r_{12}\rho_{23} e^{i(\phi_{23}+2\beta)}}. \tag{8}$$

For a normal angle of incidence, the reflectivity is therefore given by

$$R = |r|^2 = \frac{r_{12}^2 + \rho_{23}^2 + 2r_{12}\rho_{23}\cos(\phi_{23}+2\beta)}{1 + r_{12}^2\rho_{23}^2 + 2r_{12}\rho_{23}\cos(\phi_{23}+2\beta)}, \tag{9}$$

where $\theta_1 = \theta_2 = 0$ and therefore $\cos\theta_1 = \cos\theta_2 = 1$. The spectral reflectivity from our FDTD-based modelling matches this analytical result, as shown in Supplementary Fig. 15a.

When an electromagnetic wave interacts with a small spherical particle, "Lorenz–Mie theory" provides a rigorous analytical solution for Maxwell's equations. The scattering efficiency factor Q_{sca} of a single sphere is calculated via scattering cross-section C_{sca} ([7], Chapter 11)

$$Q_{\text{sca}} = \frac{C_{\text{sca}}}{\pi a^2} = \frac{2}{x^2} \sum_{n=1}^{\infty} (2n+1) \left(\left|a_n^2\right| + \left|b_n^2\right| \right), \tag{10}$$

where a is sphere radius ($D/2$), a_n and b_n are Mie scattering coefficients calculated from the complex refractive index of the sphere and Riccati–Bessel functions. Supplementary Fig. 15b shows a perfect agreement between our FDTD simulation and Lorenz–Mie theory for the scattering efficiency of a single silica sphere.

Furthermore, our simulated results agree relatively well with the measured experimental spectral absorptance of single-layer structure and multilayer structures on tungsten [5], as shown in Supplementary Fig. 15c,d. Note that experimental results also have intrinsic inaccuracies due to tolerances in manufacturing and materials impurities. This validation reinforces the confidence in our FDTD modelling.

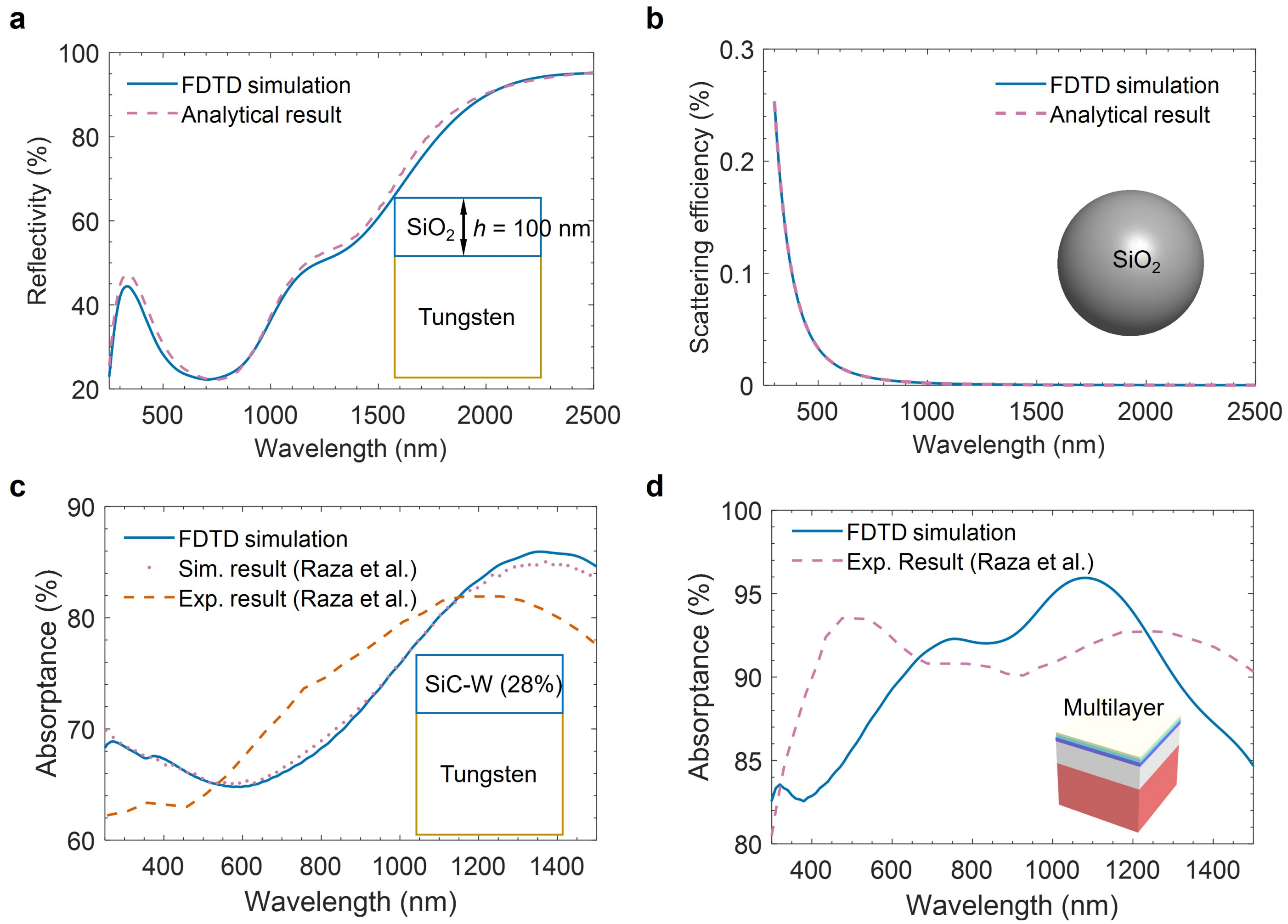

Supplementary Figure 15 | Verification and validation of CEM modelling. a, Verification of the simulated reflectivity of 100 nm thickness silica matrix on tungsten by comparison with the analytical solution in Supplementary Eq. (9). **b,** Verification of the simulated scattering efficiency of a single 100 nm diameter silica sphere by comparing with the analytical solution in Supplementary Eq. (10). **c,** Validation of the simulated absorptance of a single 33 nm SiC-W layer on tungsten with the experimental result in [5] (their simulated result is also plotted in green dotted line as a reference). **d,** Validation of the simulated absorptance of the multilayer absorber which was used in this paper with the experimental result in [5].

Supplementary Note 2:
Scalability experiment for drone-assisted nanolayer deposition

Drone-assisted deposition tests were conducted to demonstrate the scalability of the nanolayer on surface areas exceeding $1\,\mathrm{m}^2$. Here, hotplates that can heat samples up to 350 °C were prepared to mimic a receiver surface. In real concentrating solar power (CSP) plants, the temperature of the receiver surface can be controlled by circulating the heat transfer fluid (premixed from both hot and cold tanks) through the receiver, without using concentrated sunlight from the heliostats (because this light would affect the drone). A camera and spray nozzle specifically designed for mid-air spraying were attached to the drone. The drone and nozzle were controlled independently and remotely by a pilot and nozzle operator, respectively, but an autonomous arrangement with preset paths and spray regimes could also be implemented. For safety and accurate application of the nanolayer, the drone was equipped with a front-facing rangefinder (distance sensor) to maintain a distance of approximately 0.5 m while in flight from the tip of the spray nozzle top to the target surface. Wind conditions should be considered when deciding the spray distance, with longer distances achievable in stagnant air. The spray nozzle was equipped with a two-axis gimbal.

The flight control system performed tilt-angle stabilisation and spray-trajectory-angle-error correction. As a result, even when the drone was disturbed due to wind, the distance from the spray nozzle to the target surface, as well as the drone orientation, were both kept nearly constant. This stabilisation approach allowed an efficient and continuous deposition of the nanolayer. The nanolayer solution was applied twice, with each spray lasting for 10 s. During each flight, while the pilot kept the position of the drone steady in mid-air, the nozzle operator remotely manipulated the direction of the spray nozzle as well as the open/close state of the ink valve to ensure uniform application of each coat over the target surface.

In the first set of experiments, hotplates were placed on a $1.8\,\mathrm{m} \times 1.8\,\mathrm{m}$ backboard with diagonal and vertical arrangements (Supplementary Fig. 16a,b). Two separate hotplates allowed testing of the potential detrimental effect when coating an adjacent region with a volatile gas (byproduct) that could contaminate previously coated surfaces (and hence affect their absorptance). Our tests show that such a contamination did not exist because there is a negligible difference in the measured spectral absorptance ($< 0.1\%$) for all samples deposited here compared to the results shown in Fig. 5b,e. Curved samples were heated by tubular geometries resembling the surface of a central tower CSP receiver (Supplementary Fig. 16c). Large surfaces were also coated, e.g. $1\,\mathrm{m}^2$ cut of a bamboo fence, tubular arrangement is also similar to central tower solar thermal receivers [8] (Supplementary Fig. 16d). We succeeded in coating all solar absorbers on both planar and curved coupons (Supplementary Fig. 16e). A detailed scanning electron microscopy (SEM) analysis show that the quality of the drone-deposited nanolayer is the

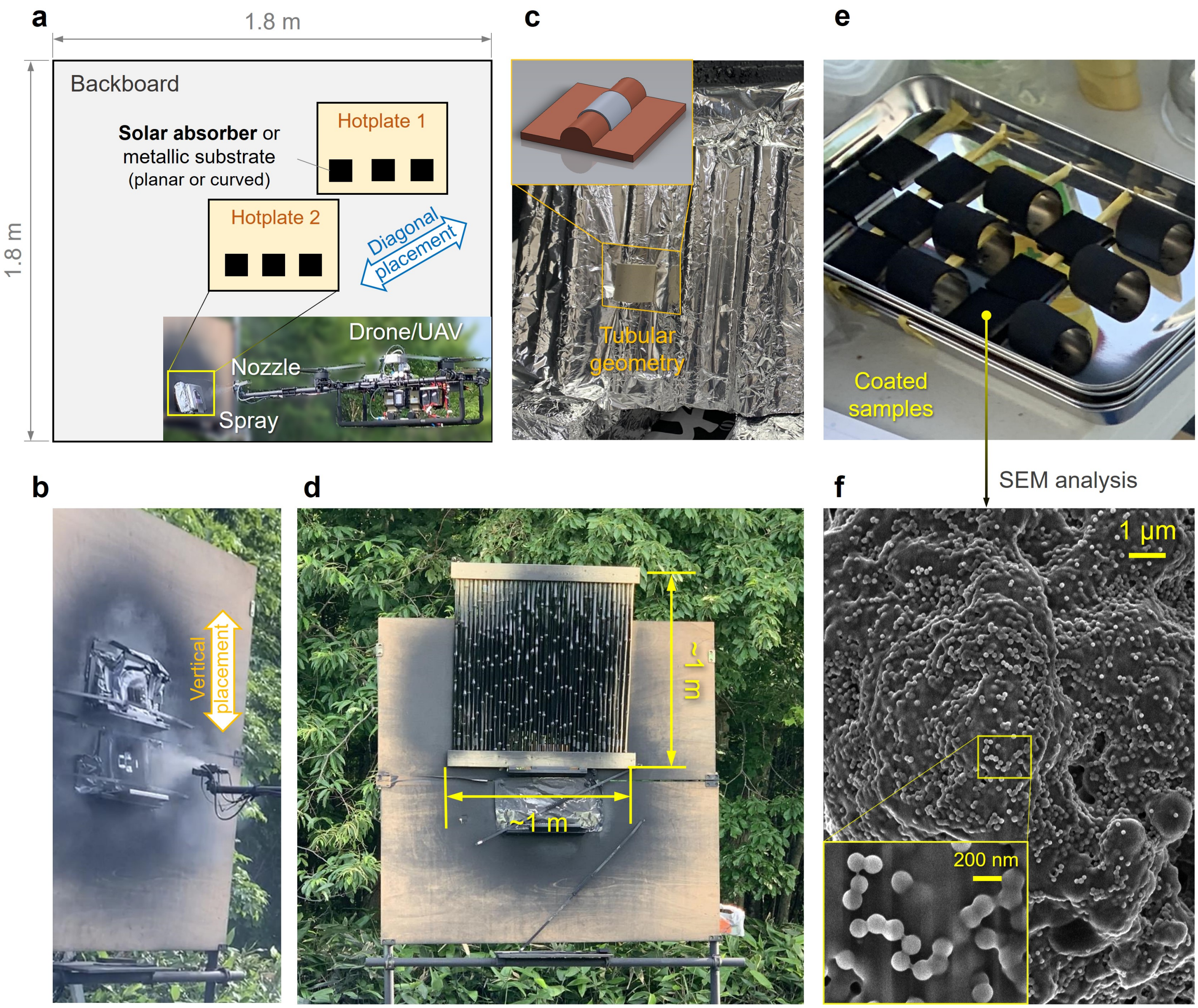

Supplementary Figure 16 | Photos of drone-assisted deposition experiment: procedure and analysis. **a,** Schematic diagram of drone-assisted deposition with diagonally placed hotplates on the backboard. **b,** Drone-assisted deposition of nanolayer on planar and curved surfaces with hotplates having a vertical arrangement. **c,** Tubular surface geometry for heating curved samples. **d,** Drone-assisted deposition on a $1\,m^2$ bamboo fence section. **e,** Samples with nanolayer from drone-deposition experiments on both planar and curved samples. **f,** SEM analysis of a drone-deposited nanolayer to confirm morphology characteristics and compare with lab-based samples (Fig. 5b).

same as for those samples produced in a lab environment, both for low and high magnifications (Supplementary Fig. 16f).

The Supplementary Movie 1 shows the deposition of coral-structured coatings on three types of surfaces. The movie is shown at a speed of ×3 and the time of each test is indicated in the lower right corner of the movie. The deposition of the nanolayer on the coral-structured coating uses the same drone and spray pattern. The deposition of a solar absorber was chosen in this movie because its black surface is more clearly visible.

1. Flat coupons (00'05"): Flat coupons were placed and fixed on the hotplates. The drone was used to spray the nanolayer on coral-structured and Pyromark 2500 absorber coatings. This video shows a configuration where the heaters are placed diagonally. Our drone deposition follows a square-grid pattern. The metallic coupons were 30 mm × 30 mm in size and 3 mm thick.
2. Curved surfaces (00'49"): Cut tubes of 25 mm in external diameter (22.6 mm internal diameter) were placed on the curved surface whose temperature was controlled with a heater. This curved corrugation resembles the surface of a receiver. Copper was used as the material due to its high thermal conductivity and heat storage capability (density × heat capacity).
3. Large surfaces (01'10"): A cut of a bamboo fence of approximately 1 m^2 in surface area was coated with the nanolayer using the same deposition parameters as the flat and curved samples. This surface area can be covered within a couple of minutes (shown in Supplementary Fig. 16d).

Supplementary Note 3:
High-temperature thermal emittance measurements

To study the potential impact of the nanolayer on the thermal emittance, we considered a solar absorber coating whose main characteristic is the presence of many micropores. These micropores resemble cylindrical holes that are known to enhance both light absorption and thermal emission [9]. This solar absorber coating is the same "base layer" described in [10]. SEM images of the baseline absorber with (top) and without (bottom) nanolayer are shown in Supplementary Fig. 17a for low (left) and high (right) magnifications. After applying the nanolayer, we confirmed a spectral absorption enhancement in pristine condition, as shown in Supplementary Fig. 17b. Here, additional curing was applied to stabilise the coating (a stable coating is a requirement for the high-temperature thermal emission measurements reported in this technical note). The pristine condition was obtained after curing at a temperature (700 °C) and time (2 h) greater than the emittance measurement condition (maximum measurement temperature was 600 °C), which rules out morphology and composition changes during the measurement (the testing time is less than 1 h).

A custom-made high-temperature Fourier transform infrared (FTIR) spectroscopy equipment [11] (IRTracer-100, Shimadzu) was utilised to measure the emissive power in the spectral range of 3 µm to 20 µm for different surface temperatures. The FTIR setup includes a blackbody furnace, a sample holder, heaters and an optical unit. More details on our custom-made setup can be found in [11]. The spectral normal emittance ε_λ is defined as

$$\varepsilon_\lambda = \frac{E_\lambda}{E_{\mathrm{b},\lambda}}, \tag{11}$$

where E_λ is the spectral normal emissive power of the sample and $E_{\mathrm{b},\lambda}$ is the spectral normal emissive power of the blackbody, both at the same temperature. Our high-temperature FTIR [11] can measure both emissive power (in arbitrary units) of a heated sample and blackbody. However, monitoring and perfectly matching the surface temperature of the nanolayer to that of the blackbody furnace is challenging (due to inevitable temperature gradients through the absorber coating). Such accurate measurements of surface temperature are out of the scope of this study. Instead, the sample heaters were set at temperatures of 400 °C, 500 °C and 600 °C and allowed sufficient time (approximately half an hour) for the temperatures to stabilise. Note that we can expect a small difference between the surface temperature of all samples because the thermal contact resistance between coupons and heater is the same (since we use the same material roughness and contact pressure) and the thermal resistance through the nanolayer is negligible (since it is thinner than 125 nm).

The spectral emissive power measurements for the case with and without nanolayer are shown in SupplementaryFig. 17c for different temperatures (which is close to the coating surface temperature). We

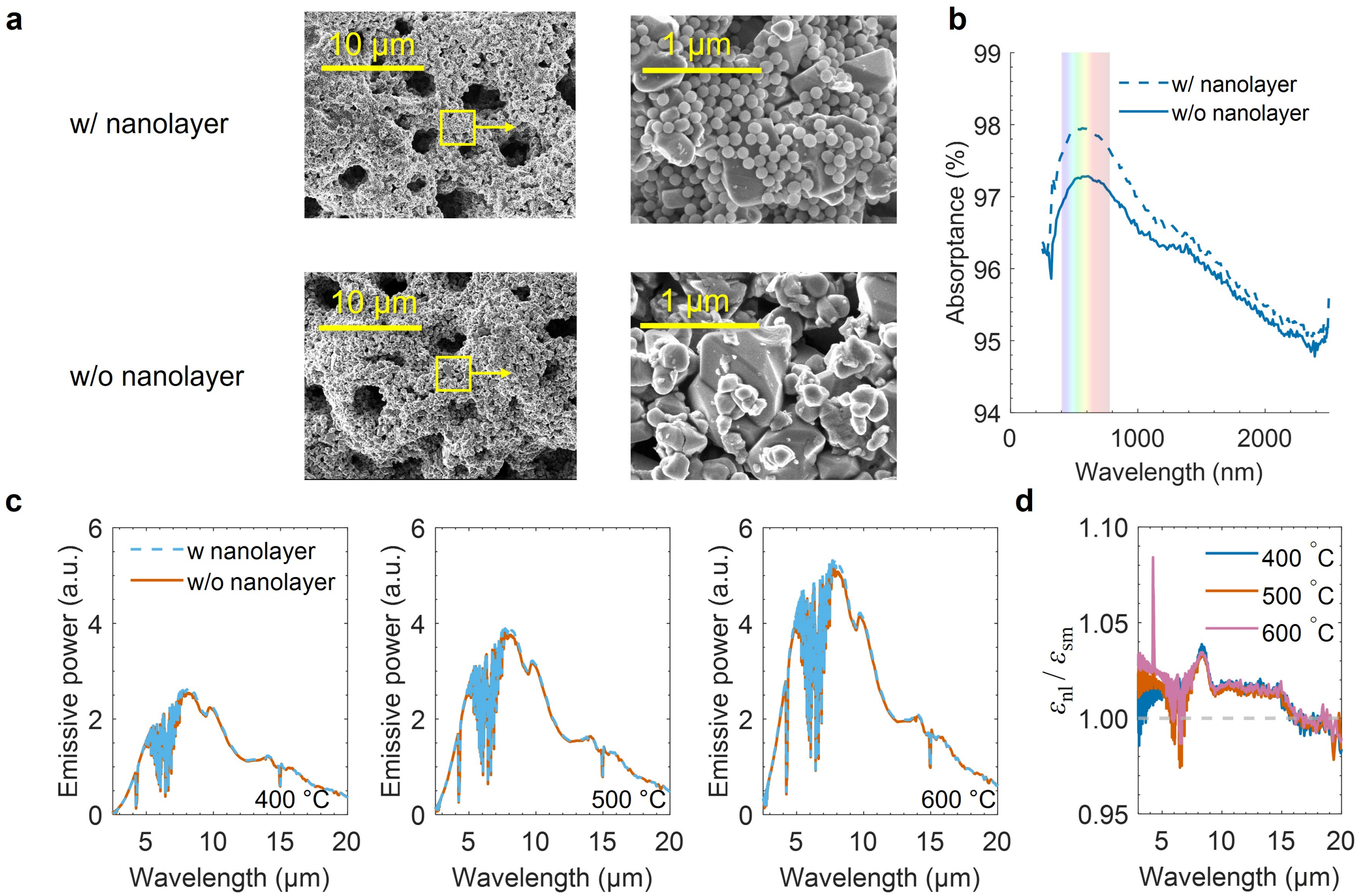

Supplementary Figure 17 | Nanolayer on absorber and spectral emissive power with and without nanolayer. **a,** SEM analysis of the underlying solar absorber with and without nanolayer. **b,** Measured spectral absorptance of the absorber with and without nanolayer. **c,** Measured emissive power signal at three heater temperatures up to 600 °C. The emissive power is measured with a custom-made high-temperature FTIR [11]. **d,** The ratio of measured thermal emittance of samples with nanolayer (ε_{nl}) and without nanolayer (ε_{sm}) at the three measured temperatures.

found that there is a small but measurable difference in the emissive power signal between samples with and without nanolayer. From Supplementary Eq. (11), the relative increase in spectral emittance due to the nanolayer can be obtained from the ratio in emissive power with nanolayer to the case without nanolayer. That is

$$\frac{\varepsilon_{\lambda,\mathrm{nl}}}{\varepsilon_{\lambda,\mathrm{sm}}} = \frac{E_{\lambda,\mathrm{nl}}}{E_{\lambda,\mathrm{sm}}}, \tag{12}$$

where the subscripts "nl" and "sm" indicate values with nanolayer and for the solar material without nanolayer, respectively. Furthermore, the spectral emittance of each surface can be weighted against the blackbody emissive power at a given temperature T to obtain the emittance ratio (relative increase of emittance with nanolayer) as follows:

$$\frac{\varepsilon_{\mathrm{nl}}}{\varepsilon_{\mathrm{sm}}} = \frac{\dfrac{1}{\sigma T^4}\displaystyle\int_0^\infty \varepsilon_{\lambda,\mathrm{nl}} E_{b,\lambda}\,\mathrm{d}\lambda}{\dfrac{1}{\sigma T^4}\displaystyle\int_0^\infty \varepsilon_{\lambda,\mathrm{sm}} E_{b,\lambda}\,\mathrm{d}\lambda} = \frac{\displaystyle\int_0^\infty E_{\lambda,\mathrm{nl}}\,\mathrm{d}\lambda}{\displaystyle\int_0^\infty E_{\lambda,\mathrm{sm}}\,\mathrm{d}\lambda}, \tag{13}$$

where the E_λ can be in arbitrary units as long as it is measured with the same device in the same configuration. The results—plotted in Supplementary Fig. 17d—lead to the following conclusions:

1. The increment in spectral thermal emittance after introducing the nanolayer is consistently below 4% (relative value) for most wavelengths. The relative increment in emittance, as per Supplementary Eq. (13), for 400 °C, 500 °C and 600 °C are $\varepsilon_{\mathrm{nl}}/\varepsilon_{\mathrm{sm}} = 1.014$, 1.013 and 1.016, respectively. That is, there is a relative increase in emittance of about 1.5%.

2. The relative increment in thermal emittance after introducing the nanolayer has a weak dependence on the surface temperature.

Here, we provide a case study using our experimentally obtained solar absorptance values and estimated emittance values, where the emittance of coral-structured coating with nanolayer is obtained from [10] and the emittance of that coral-structured coating without nanolayer by using $\varepsilon_{\mathrm{nl}}/\varepsilon_{\mathrm{sm}} = 1.015$. The figure of merit equates to the coating photo-thermal energy conversion efficiency η, which is the percentage of energy that is absorbed (α) minus the thermal energy lost by thermal emission [12] (excluding convective losses) and can be written as follows:

$$\eta = \alpha - \frac{\varepsilon\sigma(T^4 - T_{\mathrm{am}}^4)}{C q_{\mathrm{sun}}}, \tag{14}$$

where σ is the Stefan–Boltzmann constant, C is the sunlight concentration, q_{sun} is the solar flux, T is the surface temperature and T_{am} is the ambient temperature. This case study assumes $q_{\mathrm{sun}} = 980\,\mathrm{W\,m^{-2}}$, $C = 1000$ suns, and temperatures of $T = 700\,°\mathrm{C}$ and $T_{\mathrm{am}} = 25\,°\mathrm{C}$.

The results shown in Supplementary Table 1 suggest that our nanolayer has a large positive impact on

Supplementary Table 1 | Effect of nanolayer on photo-thermal energy conversion efficiency. Case study showing the change in the photo-thermal energy conversion efficiency η, which represents the figure of merit as defined in Supplementary Eq. (14). Here we consider the case of increasing the absorptance (α) and emittance (ε) *via* nanolayer deposition onto a coral-structured coating [10]. The values for the base solar material without nanolayer are indicated in blue font, while the opto-thermal properties with nanolayer values are indicated in red font.

Conditions	α (%)	$\alpha_{\mathrm{nl}} - \alpha_{\mathrm{sm}}$ (%)	ε (%)	$\varepsilon_{\mathrm{nl}} - \varepsilon_{\mathrm{sm}}$ (%)	η (%)	$\Delta\eta = \eta - \eta_{\mathrm{sm}}$ (%)
Solar material (sm) without nanolayer	96.82	–	88.18	–	92.24	–
Solar material with nanolayer (nl)	97.36	+0.54	89.52	+1.34	92.72	+0.47
increasing α; unchanged ε	97.36	+0.54	88.18	–	92.78	+0.54
unchanged α; increasing ε	96.82	–	89.52	+1.34	92.18	−0.07

the photo-thermal energy conversion efficiency η when deposited on a solar absorber coating. The positive impact on photo-thermal energy conversion efficiency η from a mild increase in solar absorptance (+0.54%) is much larger than the negative impact of a greater increase in emittance ($\Delta\varepsilon = +1.34\%$). This is because, from Supplementary Eq. (14), the high concentration of $C = 1000$ suns greatly increases the importance of solar absorptance α compared to that of thermal emittance ε.

Supplementary References

1. Hosseini, S. *et al.* Long-term thermal stability and failure mechanisms of Pyromark 2500 for high-temperature solar thermal receivers. *Solar Energy Materials and Solar Cells* **246,** 111898 (2022).
2. Rakić, A. D., Djurišić, A. B., Elazar, J. M. & Majewski, M. L. Optical properties of metallic films for vertical-cavity optoelectronic devices. *Applied Optics* **37,** 5271–5283 (1998).
3. Gao, L., Lemarchand, F. & Lequime, M. Exploitation of multiple incidences spectrometric measurements for thin film reverse engineering. *Optics Express* **20,** 15734–15751 (2012).
4. Guo, Y. *et al.* Performance enhancement of solar absorber coatings by an antireflective nanostructured layer. *AIP Conference Proceedings* **2815,** 020004 (2023).
5. Raza, A., Alketbi, A. S., Devarapalli, R., Li, H. & Zhang, T. Refractory ultrathin nanocomposite solar absorber with superior spectral selectivity and thermal stability. *Advanced Optical Materials* **8,** 2000679 (2020).
6. Born, M. & Wolf, E. *Principles of Optics: 60th Anniversary Edition* ISBN: 9781108806756 (Cambridge University Press, 2019).
7. Modest, M. *Radiative Heat Transfer* ISBN: 9780123869906 (Elsevier Science, 2013).
8. Wang, S. *et al.* Co-optimisation of the heliostat field and receiver for concentrated solar power plants. *Applied Energy* **348,** 121513 (2023).
9. Yeng, Y. X. *et al.* Enabling high-temperature nanophotonics for energy applications. *Proceedings of the National Academy of Sciences* **109,** 2280–2285 (2012).
10. Torres, J. F. *et al.* Highly efficient and durable solar thermal energy harvesting via scalable hierarchical coatings inspired by stony corals. *Energy & Environmental Science* **15,** 1893–1906 (2022).
11. Chen, J. *et al.* High-temperature optical and radiative properties of alumina–silica-based ceramic materials for solar thermal applications. *Solar Energy Materials and Solar Cells* **242,** 111710 (2022).
12. Caron, S. *et al.* A comparative analysis of opto-thermal figures of merit for high temperature solar thermal absorber coatings. *Renewable and Sustainable Energy Reviews* **154,** 111818 (2022).
