# Supplementary material for: Scalable nano-architecture for stable near-blackbody solar absorption at high temperatures
Source: Nat Commun. 2024 Jan 9;15:384. doi: 10.1038/s41467-023-44672-3 (PMC10776863; doi:10.1038/s41467-023-44672-3)
Supplement: Supplementary file 3 — Description of Additional Supplementary Files [file 41467_2023_44672_MOESM3_ESM.pdf]

Description of Additional Supplementary Files

Supplementary Movie 1: Scalability experiment for drone-assisted nanolayer deposition
